# The Book World of Medicine and Science

**Published:** 1901-11-30

**Authors:** 


### THE BOOK WORLD OF MEDICINE AND SCIENCE.

WHAT'S WHAT. A Guide for To-day to Life as it is and Things as they are. By HARRY QUILTER, M.A., Trin. Coll. Camb., Barrister-at-Law. 1902. (London: Sonnenschein and Co. Price 6s. net.)

AN interesting, amusing, and in some ways instructive encyclopædia, containing, as we are informed, 2,500 paragrams, every one of which is original and has been written since October 7th, 1900. In the construction of this work Mr. Harry Quilter has had the assistance of 51 anonymous contributors and a small staff of lady assistants, but he has himself written "more than a third of the whole book—say 350,000 words," which is no small feat considering the variety of the subjects dealt with. As for the subjects themselves they range over a very large field, and we confess we have not been able to discover the system on which the selection has been made. Of course, there is a good deal about art, literature, and journalism, but so there is about dinners, hotels, health and pleasure resorts, and all sorts of things and places that one hears talked about, while medical matters, which nowadays form the basis of so much conversation, receive ample attention—rather too ample, indeed. There is an appendix on London medical men which we think peculiarly objectionable. The names and addresses of a number of practitioners are given, and their praises are sung in a style which, we should imagine, must make some of them feel very uncomfortable; at any rate must make their friends feel very uncomfortable as to the state of medical proprieties at the present day. Of Dr. Theodore Williams we are told that he has a "wonderful ear for diagnosis," and that "we believe him to be quite trustworthy and entirely capable." "For general diseases of a grave character Sir William Broadbent, though enormously overworked is, we think, second to none." Oh, ye Royal Colleges! What think ye of all this? We should hope that Mr. Harry Quilter will have a very uncomfortable quarter of an hour next time he meets these "capable men," as he calls them. Another gentleman "is indisputably the foremost operator in such cases as fistula;" another is "a first-rate man for throat and nose diseases," and is "at the head of his profession;" while "for a kind, genial surgeon, under ordinary circumstances, we do not think anyone need want a pleasanter attendant than" Mr. Somebody else, whose wife also "is skilful and experienced." Another doctor "in all diseases relating to women" "is, we should say, the most trustworthy man in London," with "the feelings of a gentleman and the morals of a Christian;" while another doctor is recommended as having "attended one of our own children in a dangerous illness most satisfactorily." Quite like the landlady and her beds. "Aired sir? Yes sir. Why I've slep in it myself." Lest, however, we should take these recommendations too seriously, let us turn to the article on journalism. Here we see that a journalist must "be capable of writing on any subject at a moment's notice;" "must know, or seem to know, something about everything—enough to amuse and insufficient to instruct;" and must acquire the power of gripping his readers' attention "at any cost of accuracy, dignity or value." Perhaps Mr. Harry Quilter is a journalist!

DISEASES AND INJURIES OF THE TEETH INCLUDING PATHOLOGY AND TREATMENT. By MORTON SMALE, M.R.C.S., L.S.A., L.D.S., and J. F. COLYER, L.R.C.P., M.R.C.S., L.D.S. Second edition revised and enlarged by J. F. COLYER. (London: Longmans, Green & Co. 1901. Price 21s. net.)

THIS is a useful and fairly complete treatise on the diseases of the teeth and their treatment, so far as medicine, surgery, and pathology are concerned. On the other hand it does not deal with the whole range of dentistry, the details of the mechanical processes being entirely omitted. Within the limits of its title, however, it is altogether admirable, giving a more satisfactory description of the diseases and accidents to which the teeth and the structures around are liable, and of the pathology and treatment of these disorders, than any other book with which we are acquainted. It is essentially a modern book, and full value is given in it to those bacteriological researches which have had so important an influence in showing the true nature of caries and other diseases of the teeth. The chapter by Mr. Kenneth Goadby on the bacteriology of the mouth is full of information on this important subject. He shows that while in normal mouths the buccal cavity is tenanted by only a few species of micro-organisms, the number of such organisms found in mouths that are neglected, or in which carious teeth are present, is very large, especially in cases of gingivitis marginalis and in suppurative periodontitis or where there are deposits of calculus. He also points out that the environment of the individual affects the buccal flora, persons who are engaged in the care of infectious diseases often having in their mouths the organisms which are associated with the particular disease they may be tending; workers in bacteriological laboratories, for example, having pathogenic streptococci, and nurses in consumption hospitals having tubercle bacilli in their mouths. In dealing with the subject of caries the author shows the enormous and the growing frequency with which this condition is met with. He offers us, however, the comforting assurance that the figures given by certain foreign observers would seem to show that the teeth of English children compare favourably with those of German children in this respect. In discussing the causes of caries prominence is very rightly given to the relation of the teeth to one another. How far crowding of teeth goes along with the prevalence of caries we are not prepared to say, but it is impossible to doubt that any position of the teeth which interferes with the automatic cleansing arrangements by which in a state of nature accumulations of food *débris* are prevented must tend to encourage that bacterial fermentation by which caries is produced. An interesting statement is made by the authors as to the occasional spontaneous arrest of caries. Patients in whom this occurs have, they say, nearly always undergone some marked improvement in health, and although it may not be easy to see exactly how this works, the very fact that such arrest does take place in such conditions points to the importance of the state of general health as one among the causes which lead to the production as well as to the arrest of caries. The treatment of caries is very fully dealt with and a considerable space is given to the consideration of various methods adopted in "filling." Altogether the book is a very satisfactory one. While dealing with the subject in a thoroughly scientific spirit, it is full of practical information.

